# Bioinformatic Mapping of Opine-Like Zincophore Biosynthesis in Bacteria

**DOI:** 10.1128/mSystems.00554-20

**Published:** 2020-08-18

**Authors:** Jacqueline R. Morey, Thomas E. Kehl-Fie

**Affiliations:** a Department of Microbiology, University of Illinois Urbana-Champaign, Urbana, Illinois, USA; b Carl R. Woese Institute for Genomic Biology, University of Illinois Urbana-Champaign, Urbana, Illinois, USA; University of Hawaii at Manoa

**Keywords:** metallophore, metalloproteins, staphylopine, zinc

## Abstract

Zinc is an essential nutrient in biological systems due to its structural or catalytic requirement in proteins involved in diverse cellular processes. To meet this cellular demand, microbes must acquire sufficient zinc from their environment. However, many environments have low zinc availability. One of the mechanisms used by bacteria to acquire zinc is through the production of small molecules known as zincophores. Similar to bacterial siderophores used for iron uptake, zincophores are synthesized by the bacterium and exported and then reimported as zincophore-zinc complexes. Thus far, only four zincophores have been described, including two from the human pathogens Staphylococcus aureus and Pseudomonas aeruginosa, in which they play a critical role in zinc acquisition during infection, and one in a soil bacterium. To determine what other microbes may produce zincophores, we used bioinformatic analyses to identify new zincophore biosynthetic gene clusters (BGCs) and predict the diversity of molecules synthesized. Genome neighborhood network analysis identified approximately 250 unique zincophore-producing species from actinobacteria, firmicutes, proteobacteria, and fusobacteria. This indicates that zincophores are produced by diverse bacteria that inhabit a broad range of ecological niches. Many of the BGCs likely produce characterized zincophores, based on similarity to the characterized systems. However, this analysis also identified numerous BGCs that, based on the colocalization of additional modifying enzymes and sequence divergence of the biosynthetic enzymes, are likely to produce unique zincophores. Collectively, these findings provide a comprehensive understanding of the zincophore biosynthetic landscape that will be invaluable for future research on these important small molecules.

**IMPORTANCE** Bacteria must acquire essential nutrients, including zinc, from their environment. For bacterial pathogens, this necessitates overcoming the host metal-withholding response known as nutritional immunity. A novel type of zinc uptake mechanism that involves the bacterial production of a small zinc-scavenging molecule was recently described in the human pathogens Staphylococcus aureus, Pseudomonas aeruginosa, and Yersinia pestis, as well as the soil-associated bacterium Paenibacillus mucilaginosus. This suggests that zincophores may be important for zinc acquisition in diverse environments. In this study, we sought to identify other zincophore-producing bacteria using bioinformatics. We identified almost 250 unique zincophore-producing species, including human and animal pathogens, as well as isolates from soil, rhizosphere, plant, and marine habitats. Crucially, we observed diversity at the amino acid and gene organization levels, suggesting that many of these species are producing unique zincophores. Together, our findings highlight the importance of zincophores for a broad array of bacteria living in diverse environments.

## Abstract (precis)

Bacteria must acquire essential nutrients, including zinc, from their environment. For bacterial pathogens, this necessitates overcoming the host metal-withholding response known as nutritional immunity. A novel type of zinc uptake mechanism that involves the bacterial production of a small zinc-scavenging molecule was recently described in the human pathogens Staphylococcus aureus, Pseudomonas aeruginosa, and Yersinia pestis, as well as the soil-associated bacterium Paenibacillus mucilaginosus. This suggests that zincophores may be important for zinc acquisition in diverse environments. In this study, we sought to identify other zincophore-producing bacteria using bioinformatics. We identified almost 250 unique zincophore-producing species, including human and animal pathogens, as well as isolates from soil, rhizosphere, plant, and marine habitats. Crucially, we observed diversity at the amino acid and gene organization levels, suggesting that many of these species are producing unique zincophores. Together, our findings highlight the importance of zincophores for a broad array of bacteria living in diverse environments.

## INTRODUCTION

Zinc is an essential nutrient for all forms of life due to its role as a structural or catalytic cofactor for proteins involved in diverse processes, including metabolism and DNA replication (1). Between 6 and 10% of all proteins are predicted to bind zinc (2), and it is the second most utilized metal cofactor after magnesium (1). All organisms, including bacteria, must acquire sufficient zinc from their environment to meet this cellular demand. For bacterial pathogens, this necessitates scavenging zinc from host tissues; however, during infection, the vertebrate host restricts metal availability from invading pathogens in a process termed nutritional immunity (3). A key way the host restricts the availability of zinc at sites of infection is through the S100A8/S100A9 heterodimer calprotectin, which is produced by a range of host cells but predominantly by neutrophils at infection sites (4). Calprotectin restricts the availability of transition metals, including zinc, by binding them with femtomolar to picomolar affinity, thereby withholding them from invading bacteria (5–8). In an analogous situation, environmental bacteria also need to import sufficient zinc from a wide range of environments known to be zinc poor, including calcareous soils and seawater (9, 10). Furthermore, similar to vertebrate hosts, plants are also known to induce metal-withholding responses that can limit the growth of pathogenic bacteria and fungi (11). Bacteria in these environments must also compete with tens of thousands of other species in the local microbiota, as well as macrobiota including plants, for nutrients including metals (12, 13). Together, these diverse environments necessitate that bacteria possess high-affinity mechanisms that allow them to compete for zinc in order to persist in these environments. However, for many bacteria, the mechanisms they employ to acquire sufficient zinc from their local environment are poorly understood. Considering the criticality of zinc acquisition by bacteria and the importance of these species to human health, agriculture, and ecological systems, a more comprehensive picture of how diverse bacteria acquire zinc will enhance our ability to manipulate the bacterial communities in these contexts to our advantage.

To acquire sufficient zinc, most bacteria possess homologs of Znu/Adc, an ATP-binding cassette (ABC) permease system capable of directly importing labile zinc with nanomolar affinity (14). These systems are important for the virulence of numerous pathogens, including Streptococcus pneumoniae, Acinetobacter baumannii, and Salmonella enterica (15–20), which highlights the importance of high-affinity zinc import during infection. Some bacteria also encode a ZupT homolog from the ZIP zinc-transporter family, which provides additional zinc import in zinc-limiting conditions, such as during infection (21, 22). Finally, a third type of zinc-import system was recently described in bacteria that more closely resembles siderophore-mediated iron uptake (23–27). The latter machinery is commonly referred to as the Cnt system and includes a bacterially synthesized small molecule, known as a zincophore, which is exported from the cell to bind zinc, followed by import of the zincophore-zinc complex. The Cnt system was initially described in S. aureus, which also possesses an Adc homolog (24). The Cnt system is more important than Adc to the ability of S. aureus to resist calprotectin-mediated zinc sequestration *in vitro* and for virulence in a systemic mouse model of infection (24). An analogous Cnt system was subsequently described in the opportunistic human pathogen Pseudomonas aeruginosa (25, 26). In this bacterium, Cnt is required for zinc uptake in zinc-limited media and for growth in airway mucus secretions and is transcriptionally upregulated in human burn wounds (26, 28, 29). While the zincophores from both S. aureus and P. aeruginosa are important for zinc uptake by their respective species, their chemistry also permits them to bind other transition metals and facilitate their import, including iron, copper, cobalt, and nickel (23, 26). Further studies are needed to determine whether uptake of non-zinc transition metals by zincophores is physiologically relevant. This broad-spectrum metal-binding capacity has led these molecules to also be referred to as metallophores. In addition to the zincophores produced by S. aureus and P. aeruginosa, the biosynthetic pathways for zincophores from Yersinia pestis and Paenibacillus mucilaginosus have also been identified and reconstituted *in vitro*, although the physiological contribution of these zincophores to their producing species has not been elucidated (27, 30). Importantly, a limited bioinformatics study identified putative zincophore synthetic loci in a broad range of bacteria (24), suggesting that zincophores are produced by a much wider array of bacteria than the four species that have been described to date.

**FIG 1:**
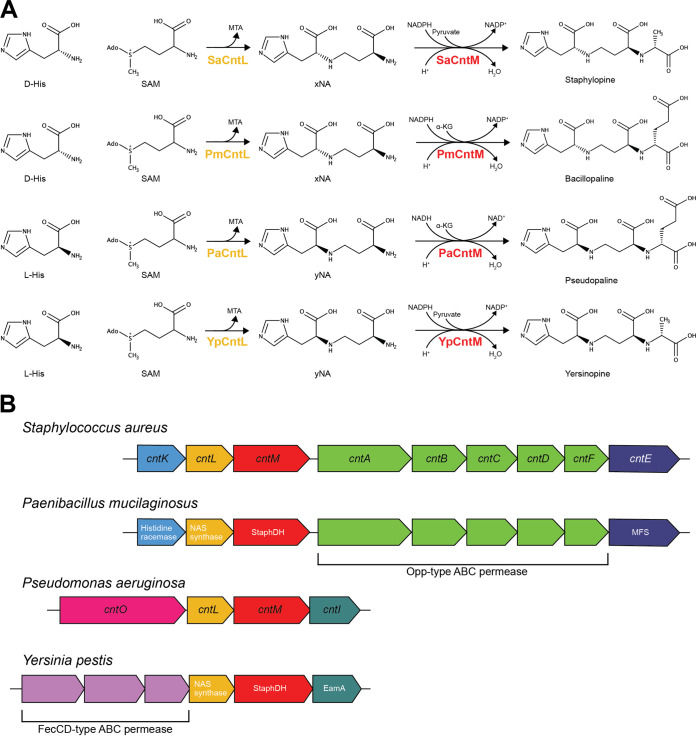
Overview of the staphylopine, bacillopaline, pseudopaline, and yersinopine zincophore systems. (A) Biosynthetic pathways of the zincophores staphylopine from S. aureus, bacillopaline from *P. mucilaginosus*, pseudopaline from P. aeruginosa, and yersinopine from Y. pestis. SAM represents *S*-adenosylmethionine, MTA is methyladenosine, and α-KG is α-ketoglutarate. The enzyme colors correspond with panel B. Note that the step catalyzed by histidine racemase (CntK) in S. aureus and *P. mucilaginosus* to convert l-His to d-His is not shown for brevity. (B) Biosynthetic gene cluster organization for the production of staphylopine, bacillopaline, pseudopaline, and yersinopine. Genes with similar functions (either predicted or experimentally determined) are colored the same, as follows: histidine racemase (light blue), NAS synthase (yellow), StaphDH (red), Opp-type ABC permease (green), MFS efflux system (dark blue), TonB-dependent OM protein (pink), EamA efflux system (teal), and FecCD-type ABC permease (purple).

The biosynthetic pathways for the characterized zincophores thus far share a core set of two to three enzymes (Fig. 1) (23, 26, 27, 30, 31). In S. aureus and *P. mucilaginosus*, the first enzyme in the pathway, a histidine racemase (CntK), converts the more abundant l-isomer of histidine (l-His) to d-His. The second enzyme, a nicotianamine synthase (NAS synthase; CntL), attaches an α-butyric acid moiety from *S*-adenosylmethionine (SAM) onto d-His to form the intermediate xNA. Finally, the third enzyme in the pathway, staphylopine dehydrogenase (StaphDH; CntM), reductively condenses xNA with an α-keto acid. In S. aureus this α-keto acid is pyruvate, compared to α-ketoglutarate (α-KG) in *P. mucilaginosus* (23, 27), resulting in the formation of the zincophores staphylopine and bacillopaline, respectively. The biosynthetic pathways for the P. aeruginosa and Y. pestis zincophores are similar but lack histidine racemase; their CntL homologs instead utilize l-His and thereby produce the alternative yNA intermediate (26, 30, 31). Like bacillopaline, CntM from P. aeruginosa (PaCntM) utilizes α-KG to form pseudopaline, while Y. pestis CntM (YpCntM) incorporates pyruvate to form yersinopine (26, 30, 31). These subtle differences in the four biosynthetic pathways (l-His versus d-His; pyruvate versus α-KG) result in the formation of four similar yet structurally unique zincophores (Fig. 1A). Notably, the zincophores produced resemble the opine and opaline compounds that are synthesized by a range of organisms, including cephalopods, yeast, and bacteria (32–34). Biosynthesis of these opine and opaline compounds is dependent on opine dehydrogenases, which catalyze the reductive condensation of an amino acid with an α-keto acid. Bacterial zincophores can therefore also be referred to as opine-like zincophores.

While only a few bacterial opine-like zincophores have been characterized to date, two general “rules” have been described for predicting the type of zincophore that will be produced based on a biosynthetic gene cluster (BGC) (27). The first of these, the presence or absence of a histidine racemase, simply determines which stereoisomer of histidine will be utilized by NAS synthase, thereby changing the stereochemistry of the histidine moiety in the zincophore. The second rule determines the α-keto acid selectivity of CntM, whereby an aspartic acid residue at position 150 in CntM (SaCntM numbering) selects for pyruvate, whereas alanine at this position selects for α-KG. Indeed, substitution of this residue in PaCntM, Ala153, with aspartic acid is sufficient to abolish activity with α-KG while increasing activity with pyruvate (27). Similarly, the equivalent substitution in SaCntM showed reduced activity with pyruvate but increased activity with α-KG, such that both α-keto acids were similarly preferred by the enzyme (27). These results indicate that the amino acid at this position plays an important role in tuning the α-keto acid preference of the enzyme but that additional residues also likely contribute to this. Together, these two simple rules are sufficient to predict the differences between staphylopine, pseudopaline, yersinopine, and bacillopaline. However, it is unclear how applicable these rules will be to newly discovered zincophore synthesis loci as a result of our currently incomplete understanding of the zincophore biosynthetic landscape. In particular, it is possible that α-keto acids other than pyruvate or α-KG may be preferred by CntM homologs from other bacteria or that additional modifying enzymes may be present that produce zincophores of greater complexity, as suggested in a limited previous bioinformatic analysis (24). Furthermore, this previous analysis was performed prior to the discovery of structural variation in zincophores and the prediction rules described above and therefore did not take into account the possibility of zincophore BGC diversity correlating with zincophore structural diversity. A more expansive and detailed bioinformatic analysis is required to determine how these rules apply to a broader range of predicted zincophore-producing bacteria and to determine whether additional rules are required to account for any additional diversity that is identified.

To expand understanding of bacterial opine-like zincophore diversity, a bioinformatics approach that leveraged a combination of sequence similarity networks (SSNs) and genome neighborhood network (GNN) analysis was employed, using CntM as a handle. This analysis identified 242 unique species possessing CntM homologs from four distinct phyla (*Firmicutes*, *Actinobacteria*, *Proteobacteria*, and *Fusobacteria*), of which the majority were part of predicted zincophore BGCs. The SSN of CntM revealed 10 distinct clusters, of which four contained a characterized zincophore system. Diversity in colocalized import and export machinery was present both within and between the SSN clusters. Several BGCs also encoded predicted modifying enzymes whose functions in zincophore biosynthesis are unknown. Furthermore, we observed greater diversity in the conserved CntM residue that determines α-keto acid selectivity than previously described. These findings suggest that zincophores are produced by a wide array of bacteria and that some of these bacteria likely produce structurally distinct zincophores that do not resemble the four variants that have been characterized thus far. Collectively, these analyses greatly increase our understanding of the biosynthetic landscape of bacterial zincophores and provide a foundation to guide future studies of this recently described class of zinc-import small molecules.

## RESULTS

### CntM homologs are present in a diverse array of bacteria.

To identify all sequenced genomes that feature predicted opine-like zincophore BGCs, we generated an SSN of CntM homologs, obtained by BLAST using the S. aureus CntM sequence as our query. CntM was chosen to base the network on as it is required to catalyze the final step in opine-like zincophore biosynthesis and is therefore a defining feature of opine-like zincophore BGCs. Our search query returned 387 sequences after filtering for length, representing 242 unique species (see Table S1 in the supplemental material). With the exception of a single sequence from a eukaryote (A0A0J7K1C0; *Lasius niger, i.e.*, black garden ant), all of the sequences were of bacterial origin.

10.1128/mSystems.00554-20.4 TABLE S1 CntM-containing bacteria identified in the CntM SSN. Download Table S1, XLSX file, 0.02 MB. Copyright © 2020 Morey and Kehl-Fie. 2020 Morey and Kehl-Fie This content is distributed under the terms of the Creative Commons Attribution 4.0 International license.

**FIG 2:**
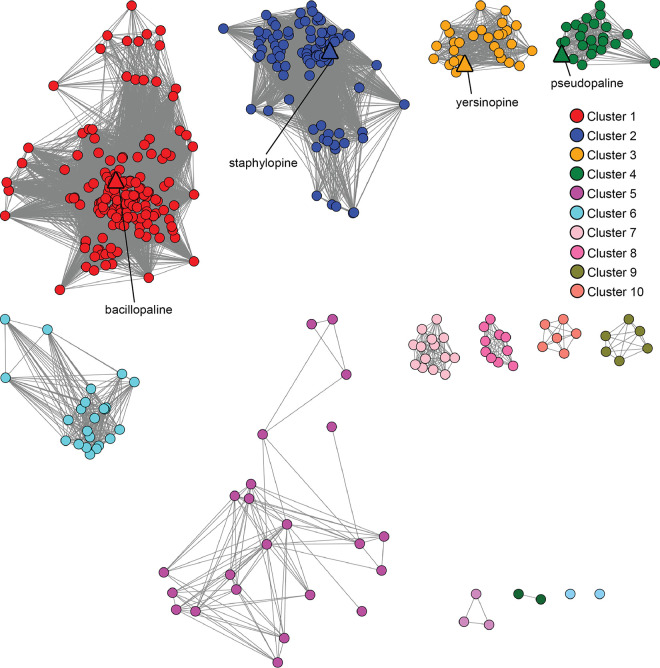
Sequence similarity network of CntM homologs. The CntM sequence from S. aureus strain Newman (UniProt ID A0A0H3KAE6) was used as a BLAST input to identify other CntM homologs. Sequences were analyzed by EFI-EST (61, 62) using an alignment score of 127, corresponding to ∼50% sequence identity, and visualized using Cytoscape (63, 64). Nodes are colored by cluster as follows: cluster 1 (red), cluster 2 (blue), cluster 3 (orange), cluster 4 (green), cluster 5 (magenta), cluster 6 (cyan), cluster 7 (pink), cluster 8 (hot pink), cluster 9 (olive), and cluster 10 (salmon). Characterized zincophores are indicated by triangles.

**FIG 3:**
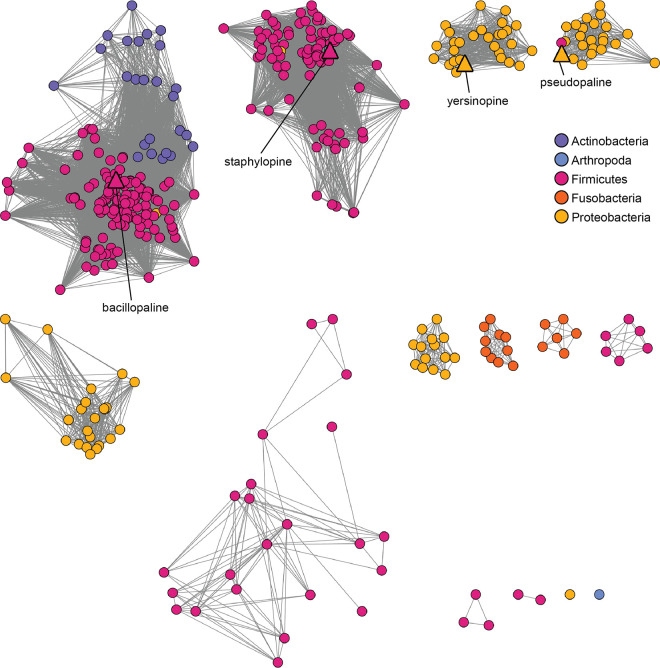
Bacterial CntM homologs are restricted to four phyla. Using the CntM SSN from Fig. 2, nodes were colored by phyla: *Actinobacteria* (purple), *Arthropoda* (blue), *Firmicutes* (pink), *Fusobacteria* (orange), and *Proteobacteria* (yellow). Characterized zincophores are indicated by triangles.

A range of threshold alignment score values were examined for fractionating the CntM sequences (see Fig. S1 in the supplemental material). We hypothesized that it would be possible to find an alignment score at which the four characterized CntM sequences clustered separately, based on their different substrate selectivity (x-NA or y-NA; pyruvate or α-KG), as a function of their sequence divergence. Using an alignment score of 127, which corresponded to ∼50% sequence identity, separate clustering of the four characterized CntM sequences was achieved (Fig. 2). SaCntM, YpCntM, and PaCntM are located in clusters comprised predominantly of members from the same genus, i.e., staphylococci for SaCntM in cluster 2, yersiniae for YpCntM in cluster 3, and pseudomonads for PaCntM in cluster 4. In contrast, PmCntM is located in cluster 1, the largest and most diverse cluster, which is comprised of species from *Bacillales*, specifically *Bacillaceae* and *Paenibacillaceae*, and *Actinobacteria*. The diverse members of cluster 1 can be separated by phylogeny (see Fig. S1E); however, cluster 1 likely represents an isofunctional set of genes based on GNN analysis (see below). The remaining clusters, which do not contain a characterized CntM homolog, are predominantly comprised of bacilli (cluster 5), vibrios (clusters 6 and 7), fusobacteria (clusters 8 and 10), and clostridia (cluster 9). Two additional clusters were observed but these contained three or fewer sequences and therefore were not considered further in our analysis. Overall, CntM homologs were observed in only four bacterial phyla: *Firmicutes*, *Proteobacteria*, *Actinobacteria*, and *Fusobacteria* (Fig. 3). To further explore the bacterial diversity represented in the network, the SSN was examined at the level of class and order, but this failed to reveal further diversity within clusters (see Fig. S2). Together, these findings indicate that CntM homologs are restricted to only four phyla, yet are produced by a wide variety of bacteria, including genera and species that have not previously been predicted to be zincophore producers.

10.1128/mSystems.00554-20.1 FIG S1 CntM SSN at different alignment scores. The CntM SSN from Fig. 2 was generated using different alignment scores, representing lower and higher fractionation than that observed in Fig. 2, with coloring from the SSN in Fig. 2 used as follows: cluster 1 (red), cluster 2 (blue), cluster 3 (orange), cluster 4 (green), cluster 5 (magenta), cluster 6 (cyan), cluster 7 (pink), cluster 8 (hot pink), cluster 9 (olive), and cluster 10 (salmon). Characterized zincophores are indicated by triangles (red, bacillopaline; blue, staphylopine; orange, yersinopine; green, pseudopaline). The alignment scores used for fractionation of the network were 87 (∼40% sequence identity) (A), 110 (∼45% sequence identity) (B), 145 (∼55% sequence identity) (C), 158 (∼60% sequence identity), (D) and 174 (∼65% sequence identity) (E). Download FIG S1, TIF file, 4.7 MB. Copyright © 2020 Morey and Kehl-Fie. 2020 Morey and Kehl-Fie This content is distributed under the terms of the Creative Commons Attribution 4.0 International license.

10.1128/mSystems.00554-20.2 FIG S2 Distribution of CntM sequences from bacteria at the class and order levels. Using the CntM SSN from Fig. 2, nodes were colored by class (A) and order (B). See the legend for the node coloring. Characterized zincophores are indicated by triangles. Download FIG S2, TIF file, 4.9 MB. Copyright © 2020 Morey and Kehl-Fie. 2020 Morey and Kehl-Fie This content is distributed under the terms of the Creative Commons Attribution 4.0 International license.

### CntM is co-occurrent with predicted zincophore BGCs.

**FIG 4:**
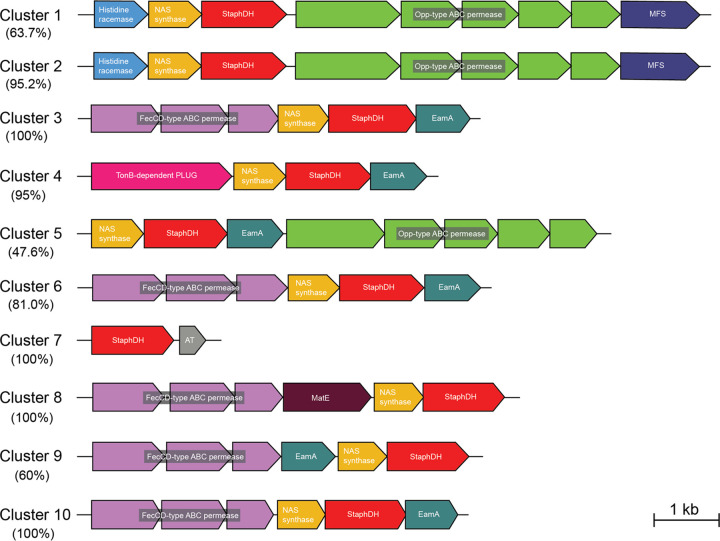
Representative gene arrangement of CntM-containing operons identified in the CntM SSN. A schematic overview of the predominant gene arrangement for CntM-containing gene clusters in clusters 1 to 10 from the CntM SSN. Genes with similar function (either predicted or experimentally determined) are colored the same. AT, acetyltransferase. The percentage of operons within the cluster that resemble the representative genome organization is reported for each cluster. Genes are drawn to scale.

While CntM homologs were used to identify potential zincophore BGCs, it is possible that some CntM homologs may be encoded within non-zincophore BGC contexts and serve a different biological function. Therefore, the colocalization of additional genes was also evaluated to assess whether the CntM sequences were part of zincophore BGCs. The four zincophore systems that have been characterized to date have in common an NAS synthase (*cntL*) encoded directly upstream of *cntM*, in addition to encoding import and export machinery within six open reading frames (ORFs) of *cntM* (23, 24, 26, 31). To determine whether the CntM homologs identified in the SSN above were part of putative zincophore BGCs, we generated a genome neighborhood network (GNN) to interrogate their local genome contexts. In all clusters except cluster 7, an ORF encoding a predicted NAS synthase homolog was observed directly upstream of *cntM* (Fig. 4). Furthermore, with the exception of cluster 7, all of the sequences encoding CntM homologs co-occurred with predicted import and efflux systems within six ORFs, consistent with the four characterized BGCs. The types of transporters localized to the zincophore BGCs are discussed in greater detail below. Together, these observations of the co-occurrent genes suggest that all clusters except cluster 7 contain *bona fide* zincophore BGCs.

In cluster 7, which is comprised entirely of *Vibrio* species, the sequence encoding CntM is always directly followed by an ORF that encodes an InterPro family IPR016181 acyl coenzyme A (acyl-CoA) *N*-acyltransferase member. As mentioned above, no predicted import or export systems are encoded within six ORFs on either side of *cntM*, and this held true even when this window was expanded to 10 ORFs on either side. The cluster 7 CntM homologs therefore do not resemble zincophore BGCs. Intriguingly, when the *Vibrio* species represented in cluster 7 were compared with those in cluster 6, we found that these largely overlapped, with only two species, Vibrio atlanticus and *Vibrio* sp. strain 10N.222.47.A9, in cluster 7 that were not also represented in cluster 6. The reverse was not true, since ∼50% of cluster 6 species were not also found in cluster 7. We therefore name the orphan CntM homolog in these *Vibrio* species CntM2, as it is almost always in addition to the primary CntM sequence located within the zincophore BGC. Collectively, these findings indicate that genes encoding CntM homologs are almost always associated with zincophore BGCs, as determined by genomic colocalization of their coding sequence with a predicted NAS synthase and import and efflux systems. However, some *Vibrio* species appear to have a second CntM homolog encoded elsewhere in the genome.

### Zincophore BGCs can encode additional potential modifying enzymes.

In addition to the core CntL and CntM enzymes, both staphylopine and bacillopaline require a histidine racemase, CntK, to first convert l-His to d-His for CntL to use as a substrate (23, 27, 30). A predicted histidine racemase was identified directly upstream of *cntL* in the vast majority of zincophore BGCs from clusters 1 and 2 (Fig. 4; Fig. S3), which suggests that these zincophore BGCs likely produce zincophores utilizing d-His, similar to staphylopine and bacillopaline. Interestingly, a number of BGCs in clusters 1, 5, and 6 were also observed to encode predicted transferases in their BGCs that were in addition to their core CntL and CntM enzymes. A predicted transferase was considered to be a part of a zincophore BGC if it was encoded within seven ORFs of CntM on the same strand. Of the 16 BGCs with additional predicted transferases from cluster 1, eight are predicted to encode a methyltransferase (from Pfam family PF13649), and eight have a predicted acetyltransferase (from either PF13673, PF00583, or PF13302). One of these loci, from *Paenibacillus* sp. strain FSL A5-0031 (CntM UniProt ID A0A1R1AQI4), also encodes an additional *cntL* homolog and a predicted transport system upstream of the zincophore BGC. In cluster 5, six BGCs contain a predicted methyltransferase (from either PF13489 or PF13847), whereas in cluster 6 a predicted acetyltransferase (PF13673) was observed in four BGCs. Two of these also encoded a predicted methyltransferase (PF13649) upstream. The role of these predicted transferase enzymes within the BGCs is not clear, but one hypothesis is that these additional enzymes are making further modifications to the zincophores produced by these species, leading to greater structural diversity overall.

10.1128/mSystems.00554-20.3 FIG S3 CntM-containing gene clusters featuring a predicted histidine racemase (related to Fig. 2). Using the CntM SSN from Fig. 2, nodes were colored purple if a predicted histidine racemase was identified two ORFs upstream of the CntM sequences, based on the CntM GNN and further SSN analysis. Gene clusters lacking a predicted histidine racemase were colored blue. Characterized zincophores are indicated by triangles. Download FIG S3, TIF file, 4.2 MB. Copyright © 2020 Morey and Kehl-Fie. 2020 Morey and Kehl-Fie This content is distributed under the terms of the Creative Commons Attribution 4.0 International license.

### A restricted set of transporters is associated with zincophore BGCs.

The BGCs for the characterized zincophores to date (staphylopine, pseudopaline, yersinopine, and bacillopaline) produce structurally distinct zincophores and also diverge in their organization, particularly in terms of the import and export systems they encode (Fig. 1). For example, the S. aureus and *P. mucilaginosus* zincophore BGCs encode a major facilitator superfamily (MFS) efflux system, while P. aeruginosa and Y. pestis encode an EamA system. This led to the question of how these two types of efflux systems were distributed in the zincophore BGCs identified in the network analysis. The majority of zincophore BGCs encoded either a predicted MFS or EamA system, consistent with the characterized zincophore systems. The type of export system predicted was largely conserved within clusters in our SSN (Fig. 4). Most of the BGCs in cluster 1 and all of the BGCs in cluster 2 encoded predicted MFS efflux systems, consistent with these clusters containing the *P. mucilaginosus* and S. aureus systems, respectively. The exception to this is three BGCs in cluster 1 that encode a predicted EamA efflux system instead of an MFS. The remaining clusters 3 to 10 encoded a predicted EamA system within their BGCs, with two exceptions: cluster 7, which features the orphan CntM, and cluster 8, which comprises fusobacteria BGCs that encode a predicted MatE transporter instead of EamA. This represents a third type of efflux system to be associated with zincophore BGCs.

The predicted importers follow a similar distribution to the efflux systems across the SSN clusters (Fig. 4). Two types of predicted import systems were identified, both from the ABC transporter family, based on their Pfam classification. The first type, belonging to the dipeptide/oligopeptide type transporters, was observed in the zincophore BGCs from clusters 1, 2 and 5. Interestingly, the actinobacteria in cluster 1 are the exception, where 14 of 24 encode the second type of import system (FecCD), while the remaining 10 encode both dipeptide/oligopeptide and FecCD types within six ORFs of their *cntM* homolog. Of the remaining clusters, clusters 3, 6, 8, 9 and 10 encode predicted FecCD-type permeases. Of note is cluster 3, in which the BGCs from Y. pestis strains encode truncated or frameshifted FecCD systems. Consistent with previous studies, no inner membrane import system was identified in the BGCs from cluster 4, which includes the characterized BGC that produces pseudopaline, and no import system was identified in cluster 7, as mentioned above. Considered together with the distribution of predicted export systems above, these findings highlight the significant diversity in zincophore BGCs, despite a narrow range of transporter types utilized.

### The CntM residue governing α-keto acid preference is moderately conserved.

**FIG 5:**
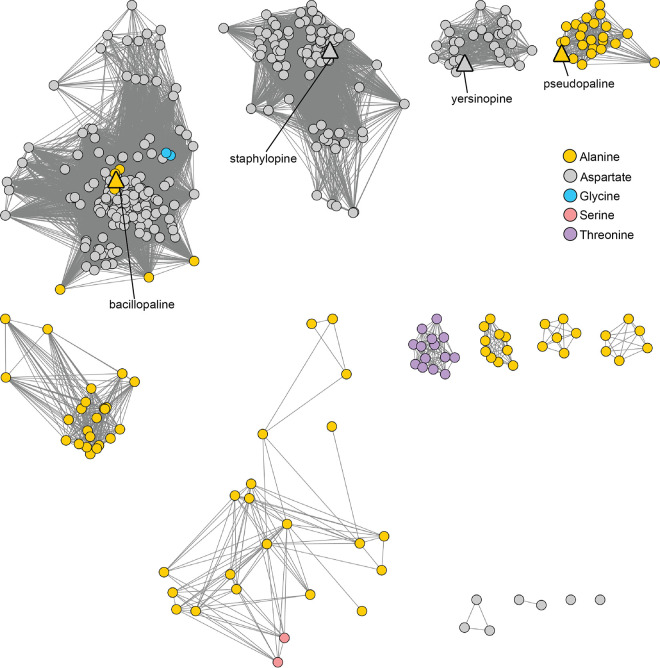
Additional diversity at CntM position 150 (SaCntM numbering). The CntM sequences from clusters 1 to 10 were analyzed by multiple sequence alignment to identify the amino acid corresponding to position 150 in S. aureus CntM, and this information was mapped back onto the CntM SSN by node color: alanine (yellow), gray (aspartate), blue (glycine), peach (serine), and lilac (threonine). Characterized zincophores are indicated by triangles.

A previous study suggested the importance of position 150 in CntM (SaCntM numbering) for governing α-keto acid selectivity (27). An aspartate at position 150 corresponds with utilization of pyruvate, as observed for SaCntM and YpCntM, whereas an alanine is selective for α-KG, as in PaCntM and PmCntM. We therefore evaluated which amino acid residue was present in this position in all of the CntM sequences and mapped this information back onto the SSN (Fig. 5). Most clusters exclusively had an alanine or an aspartate at this position in CntM. However, cluster 1 was heterogenous, with predominantly aspartate but also a number of alanine-containing sequences, including PmCntM. Strikingly, alternative amino acid residues were also identified at this position in some of the CntM sequences in the network, suggesting that these CntM homologs may be selective for alternative α-keto acids. This included two sequences with glycine in cluster 1, two with serine in cluster 5, and all 13 members of cluster 7 with a threonine at this position. Overall, these observations suggest that most of the CntM sequences identified in the network likely utilize pyruvate or α-KG, based on their having an aspartate or an alanine at position 150. However, an appreciable number of organisms are potentially utilizing different α-keto acids at this step, resulting in the synthesis of diverse zincophores.

## DISCUSSION

Zinc is an essential nutrient for all forms of life (1, 2). To acquire sufficient zinc in diverse and metal-restricted environments, bacteria use high-affinity zinc-uptake systems, including zincophore-driven systems. Zincophores are important for bacterial virulence, in some instances being the primary zinc uptake system utilized during infection, and they are likely produced by a wide array of bacteria (24). However, no studies to date have provided a comprehensive analysis of the bioinformatic space of opine-like zincophore BGCs and their diversity. The present study utilized both SSN and GNN analyses to address this, using StaphDH (CntM) as a starting point. A strength of this approach is that it enabled identification of CntM homologs without relying on automated gene annotations, which are notoriously inaccurate (35). Conversely, a significant caveat of this approach is that only zincophores that require a CntM homolog to be synthesized will be identified, meaning the diversity of bacterial zincophores may be even greater than what is suggested by the current analysis. However, given the fact that a CntM homolog is required to produce an opine-like zincophore, the sequence-driven approach employed here allows identification of all of the putative bacterial opine-like zincophore BGCs in sequenced genomes at date of manuscript preparation. CntM homologs were identified in ∼250 unique species. Of the CntM homologs that grouped into clusters in our SSN, a majority (∼95%) appeared to be part of zincophore BGCs, based on observation of a predicted NAS synthase directly upstream of the CntM homolog, as well as colocalized import and efflux systems. This indicates that CntM homologs have a biological role that is restricted to zincophore biosynthesis, which highlights the continued evolutionary pressure imposed by zinc-limited environments on bacteria to maintain zincophore biosynthesis rather than CntM undergoing functional evolution.

The current analysis identified zincophore BGCs in a broad range of bacteria, including actinobacteria, clostridia, fusobacteria, and vibrios. This observation suggests that bacteria in highly diverse environments are harnessing zincophores to meet their cellular demand for zinc. One such environment where zincophores play an important role is during infection of animal hosts, including humans. This is supported by studies demonstrating the importance of zincophores for the human pathogens S. aureus and P. aeruginosa during infection (24, 25, 28). The physiological contribution of yersinopine to Y. pestis and Y. pseudotuberculosis, the causative agents of plague and Far East scarlet-like fever, respectively (36), is less clear due to a lack of studies characterizing these systems *in vivo*. This is compounded by the fact that the Y. pestis FecCD-type import system appears to be frameshifted or truncated, suggesting that this species still produces yersinopine but cannot import it. However, it is possible that Y. pestis utilizes an alternative transporter to import yersinopine-zinc complexes (37, 38). Additional human and animal pathogenic species were identified in the network, including the opportunistic pathogen Serratia marcescens (39), and the oral pathogen Fusobacterium nucleatum (40). The presence of zincophore BGCs in an array of pathogenic bacteria suggests that zincophores are an effective mechanism used by numerous pathogens to overcome nutritional immunity, similar to what has been observed for S. aureus (24). If this is true, zincophore systems may be excellent potential targets for antimicrobial therapies. One therapeutic avenue could be to inhibit zincophore efflux, since deletion of CntE in S. aureus causes increased sensitivity to calprotectin *in vitro* and a dramatic reduction in virulence (41, 42). Similarly, in P. aeruginosa, deletion of the efflux system CntI causes a growth defect that is rescued by preventing pseudopaline synthesis, as well as causing a reduction in virulence in burn wound and lung infection models (26, 29). While the precise mechanism of toxicity has not been elucidated, it is likely that other zincophore-producing species will be similarly sensitive to loss of zincophore efflux. This is supported by the GNN analysis, in which almost all the zincophore BGCs identified included a predicted efflux system. Further studies are needed to determine how other zincophore-producing species respond to loss of zincophore efflux. Importantly, the diversity in the types of efflux systems utilized by zincophore-producing bacteria may present an opportunity to design narrow spectrum antimicrobials that target specific groups of zincophore producers via their type of efflux system. This may be particularly relevant in soil and rhizosphere contexts, where multiple zincophore-producing species utilizing different types of efflux systems, such as actinobacteria, bacilli, and pseudomonads, are likely to coexist.

In addition to the human and animal pathogens in the network, many of the zincophore BGCs were identified in environmental bacterial isolates from the rhizosphere, soil, plant tissue, and seawater. Interestingly, all of these environments can be severely zinc limited. Similar to animals, plants can withhold transition metals from pathogenic bacteria inhabiting the rhizosphere to limit their growth (11). Furthermore, the rhizosphere is thought to be nutrient limiting as a result of intense interspecies competition for nutrients between pathogenic and commensal bacterial and fungal species, as well as plants (43, 44). This is exemplified by the arsenal of siderophores produced by beneficial bacteria in the rhizosphere, which enables them to outcompete pathogenic species in that niche (45, 46). More generally, many areas of the world have soil with poor zinc availability as a result of high soil pH, the presence of organic matter that strongly chelates zinc, or high CaCO_3_, among other factors that preclude microbes from obtaining this essential nutrient (9). The present study suggests that many of these soil-dwelling species likely produce zincophores to overcome low zinc availability in these environments. Plants may also be able to use zincophores from beneficial bacteria, similar to what has been observed for bacterial siderophores (47, 48). Many plants encode an NAS synthase homolog that synthesizes a metallophore from three molecules of SAM to make nicotianamine, which is responsible for transition metal homeostasis throughout the plant (49). It is tempting to speculate that plants may be able to use the structurally similar bacterial zincophores for zinc uptake and transport. Similar to most soils, seawater is also zinc limited, particularly at its surface as a result of high demand for zinc by phytoplankton and adsorption of zinc onto sinking organic particles (10, 50). Bacteria in the ocean must compete for zinc with phytoplankton, which require a large quantity of zinc to support growth and are responsible for a significant fraction of zinc geochemical cycling (51, 52). Like their soil- and plant-associated or animal-associated counterparts, this study indicates that some ocean-dwelling species are producing zincophore-like molecules to compete for zinc in this harsh environment. Broadly, the current analysis suggests that zincophores are likely to be used by pathogenic and nonpathogenic organisms across diverse environments to obtain critical nutrients.

A significant benefit of the bioinformatics approach utilized here was the ability to interrogate how the zincophore BGCs were organized. Two main types of zincophore BGCs were observed in our GNN: (i) those that had a predicted histidine racemase, an MFS efflux system, and a dipeptide/oligopeptide-type import system, and (ii) a second type that lacked a histidine racemase but had an EamA efflux system and FecCD-type import system. This is consistent with what has been observed for the BGCs of the characterized zincophores (23, 24, 26, 27). However, some notable subtypes were also observed, including some actinomycetes that encoded a predicted histidine racemase and MFS efflux system with a FecCD homolog, rather than a dipeptide/oligopeptide system, or encoded both a FecCD and dipeptide/oligopeptide-type importer. The actinomycetes therefore broke the general rule observed where the presence of a predicted histidine racemase co-occurred with a dipeptide/oligopeptide-type importer. Interestingly, the actinomycetes that encoded only FecCD were the same species that encoded an additional predicted methyltransferase, which may make further modifications to the zincophore by adding one or several R groups to the molecule to form a bulkier product. The archetypal FecCD homolog from E. coli, in conjunction with the solute-binding protein FecB, imports an array of iron-organic acid conjugates that are of comparable size to zincophores (53). The FecCD-type systems in the actinomycete zincophore BGCs are therefore potentially better suited for importing the predicted bulkier zincophores produced by these BGCs.

One of the most intriguing finds from our analysis was the discovery of numerous *Vibrio* species that encoded a second, seemingly orphan CntM homolog (CntM2), which co-occurs with a predicted acetyltransferase downstream. Predicting the chemistry performed by CntM2 based on sequence alone is challenging. CntM and CntM2 from the same species share ∼40% sequence identity and ∼60% similarity, which suggests a conserved function. By comparison, SaCntM and YpCntM, which both utilize pyruvate but differ in their selectivity for xNA or yNA, share 26.5% sequence identity and 45.6% similarity. Furthermore, CntM2 has a threonine at the conserved position known to govern α-keto acid preference in the characterized CntM enzymes, which suggests that it may use a different α-keto acid other than pyruvate or α-KG. To investigate this further, CntM2 was compared to characterized opine dehydrogenases not involved in zincophore biosynthesis, including octopine dehydrogenase from the king scallop *Pecten maximus*, saccharopine dehydrogenase from Saccharomyces cerevisiae, and opine dehydrogenase from *Arthrobacter* sp. strain 1C (ArODH) (33, 54–57). Of these, only ArODH has a threonine at the same position as CntM2. ArODH has broad substrate specificity, showing robust enzyme activity with hydrophobic amino acids and lower-molecular-weight α-keto acids such as pyruvate, oxaloacetate, glyoxylate, and α-ketobutyrate, although l-phenylalanine and pyruvate are described as its native substrates (56, 58). However, the amino acid residues involved in substrate recognition have not been identified, which makes it challenging to understand how substrate selectivity in this enzyme might also apply to CntM2. Despite this challenge, it suggests that CntM2 may be able to use a broader range of substrates, including α-keto acids and amino acids, similar to what is observed for ArODH. Under certain environmental conditions or stress, CntM2 expression could be upregulated, potentially in conjunction with the downstream predicted acetyltransferase, resulting in the production of an alternative zincophore that may provide an advantage to its producing species under these conditions. *Vibrio* species are ocean isolates that have to compete with many other bacterial and eukaryotic species for the limited available zinc, especially near the ocean surface (59). Furthermore, many *Vibrio* species are also pathogens of fish and shellfish and therefore must scavenge zinc from infected host tissues. In these environments, the ability to produce an expanded repertoire of zincophores may be invaluable to gain an edge over competitors or to strip zinc from the host organism during infection.

The current determination of the zincophore biosynthetic space has revealed that zincophores are produced more widely than previously thought, by bacteria inhabiting zinc-limited but diverse environments. Through our analysis, numerous zincophore BGCs have been identified that most likely produce novel zincophore structures, which should therefore be a high priority for future studies. Based on the current findings, continued zincophore research is likely to have important ramifications for a range of different fields, including human and animal health through improved antimicrobials against zincophore-producing pathogens, improved plant and soil health (including a greater understanding of how zincophore production by beneficial and pathogenic bacteria influences plant growth), and marine ecology and aquaculture industries, through an enhanced understanding of how competition for zinc shapes population dynamics of different species of bacteria in marine environments.

## MATERIALS AND METHODS

### Construction of CntM SSN and GNN.

To identify sequenced genomes encoding CntM homologs, the S. aureus Newman CntM sequence (UniProt ID A0A0H3KAE6) was used as an input query for BLAST via the EFI-EST web tools (60–62) using the UniProt 2020-01/InterPro 78 releases, resulting in 414 sequences. These sequences were filtered for full-length proteins by applying a minimum amino acid length of 350, resulting in 387 nodes. Fractionation was performed using an alignment score of 127, corresponding with ∼50% sequence identity. All networks were visualized in Cytoscape (version 3.7.1) (63, 64). To visualize how the network changed at less stringent and more stringent thresholds, networks were created using alignment scores 87, 110, 145, 158, and 174, corresponding to sequence identities of 40, 45, 55, 60, and 65%, using the coloring from the network at alignment score 127 (see Fig. S1). The GNN of CntM was generated using the EFI-GNT web tools (60–62) with the default parameters (neighborhood size 10, 20% minimal co-occurrence lower limit). UniProt IDs for the CntM sequences in the network are listed in Table S1 in the supplemental material. For the four characterized systems indicated on the SSNs, the UniProt IDs F8FR19 (*P. mucilaginosus*), A0A0H3KAE6 (S. aureus), A0A384K9K6 (Y. pestis), and A0A509JI41 (P. aeruginosa) were used.

### Classification of zincophore BGCs.

Using the CntM GNN, the local genomic contexts of the identified CntM sequences were scrutinized to determine whether they constituted a putative zincophore BGC. The key requirements for classification as a zincophore BGC were the presence of a predicted NAS synthase directly upstream of the CntM homolog, as well as predicted import and efflux systems encoded within six ORFs of the gene encoding CntM. Prediction of a NAS synthase was based on its annotation with InterPro family IPR004298, to which the NAS synthases from the characterized zincophore producers (S. aureus, UniProt ID A0A0H3KIR0; *P. mucilaginosus*, UniProt ID F8FR18; P. aeruginosa, UniProt ID A0A0D7MUC4; and Y. pestis, UniProt ID A0A384KKI0) belong. Identification of the type of import system present was based on Pfam family annotation, whereby we designated the dipeptide/oligopeptide type of ABC permease based on the presence of a predicted transmembrane domain from PF00528 (BPD_transp_1) and/or PF12911 (OppC_N) and a solute-binding protein from PF00496 (SBP_bac_5), while the FecCD-type ABC permease was based on a predicted transmembrane domain from PF01032 (FecCD) and solute-binding protein from PF01497 (Peripla_BP_2). Both types of ABC permeases also encoded a predicted nucleotide-binding domain from PF00005 (ABC_tran). Predicted efflux systems were based on Pfam family annotation with PF07690 (MFS_1), PF00892 (EamA), and PF01554 (MatE).

### Identification of predicted histidine racemase homologs.

To identify predicted histidine racemase homologs in the zincophore BGCs, which have no Pfam or InterPro designation, the InterPro accession IDs for all the genes neighboring CntM in the GNN that lacked a Pfam were utilized to generate a new SSN using the EFI-EST web tools accession ID input, consisting of 1,112 sequences. To exclude truncated or multidomain proteins, a size range of 150 to 400 amino acids was applied, which reduced the network to 606 nodes. The network was fractionated using an alignment score of 28, corresponding with ∼30% sequence identity. This resulted in the formation of two large clusters containing 205 and 157 sequences, with numerous significantly smaller clusters. Cluster 1 contained the sequences for histidine racemase from S. aureus and *P. mucilaginosus* (UniProt IDs A0A0H3KAG1 and F8FR17, respectively). The network was then subject to GNN analysis to examine the genomic context of the sequences. The GNN for this network was generated using the EFI-GNT web tools (60–62) with the default parameters (neighborhood size 10, 20% minimal co-occurrence lower limit). All members of cluster 1 were encoded two ORFs upstream of the CntM homolog identified in the original CntM SSN, consistent with the position of the histidine racemase in S. aureus and *P. mucilaginosus* relative to CntM. The cluster 2 members were always localized directly upstream of the gene encoding CntM in each locus and all were annotated as being InterPro family IPR004298 members, consistent with them being NAS synthase homologs. The cluster 1 sequences were therefore considered to be homologs of histidine racemase. These were mapped back onto the CntM SSN, indicating that predicted histidine racemases were only found in zincophore BGCs from clusters 1 and 2 of the CntM SSN (see Fig. S3). The UniProt IDs for the histidine racemase homologs are listed in Table S2 in the supplemental material.

10.1128/mSystems.00554-20.5 TABLE S2 Predicted histidine racemases within zincophore BGCs. Download Table S2, XLSX file, 0.01 MB. Copyright © 2020 Morey and Kehl-Fie. 2020 Morey and Kehl-Fie This content is distributed under the terms of the Creative Commons Attribution 4.0 International license.

### Other protein sequence comparisons.

To identify conserved residues in CntM, the sequences from each cluster of the CntM SSN were aligned using Clustal Omega and the default ClustalW parameters (65). Pairwise sequence comparisons were performed using EMBOSS Needle with the default settings (65).

### Data availability.

The accession IDs for all of the sequences identified and analyzed in this work are listed in Table S1.
