# Supplementary figures and images for: Bioinformatic Mapping of Opine-Like Zincophore Biosynthesis in Bacteria
Source: mSystems. 2020 Aug 18;5(4):e00554-20. doi: 10.1128/mSystems.00554-20 (PMC7438024; doi:10.1128/mSystems.00554-20)

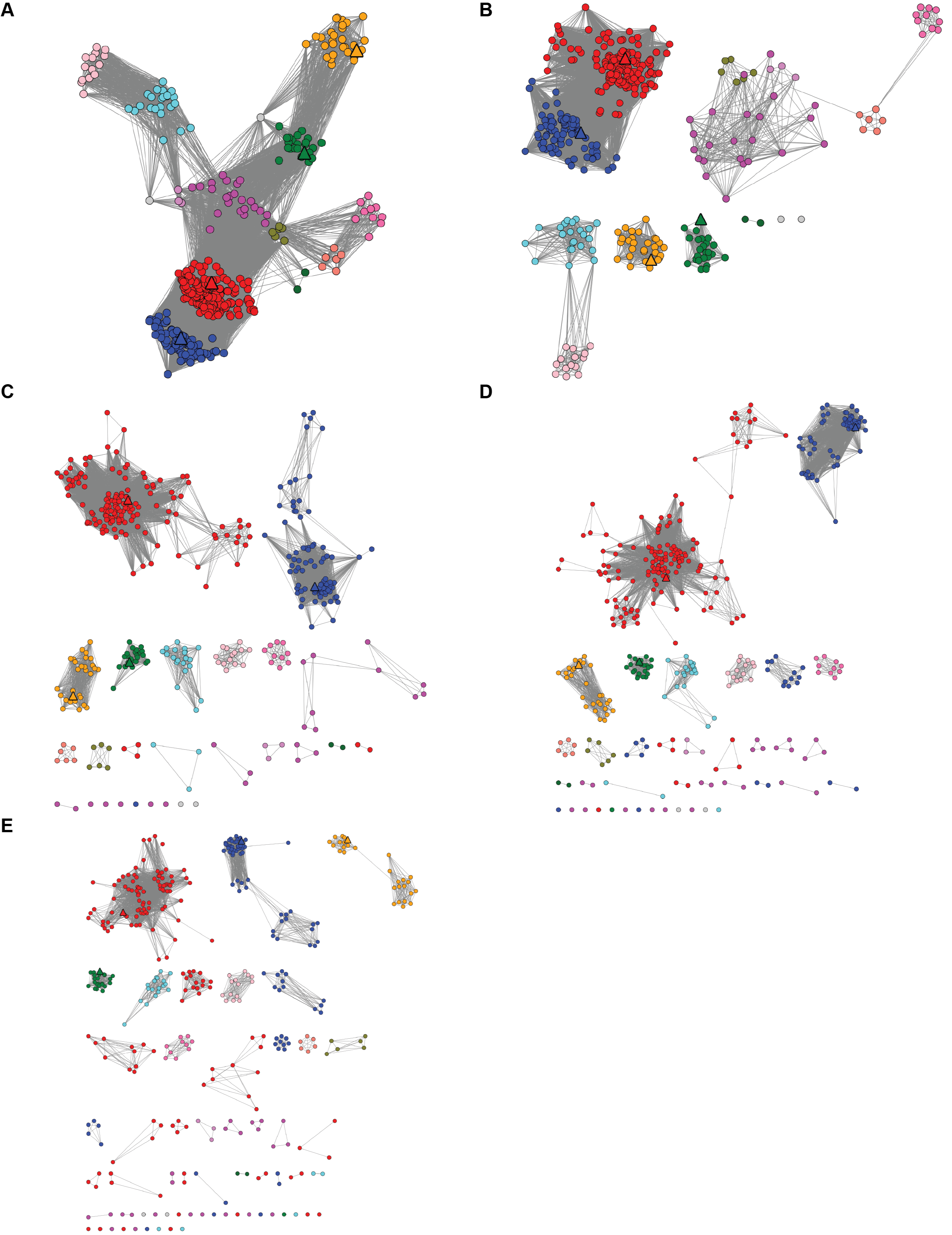

Supplement: FIG S1 [file mSystems.00554-20-sf001.tif]

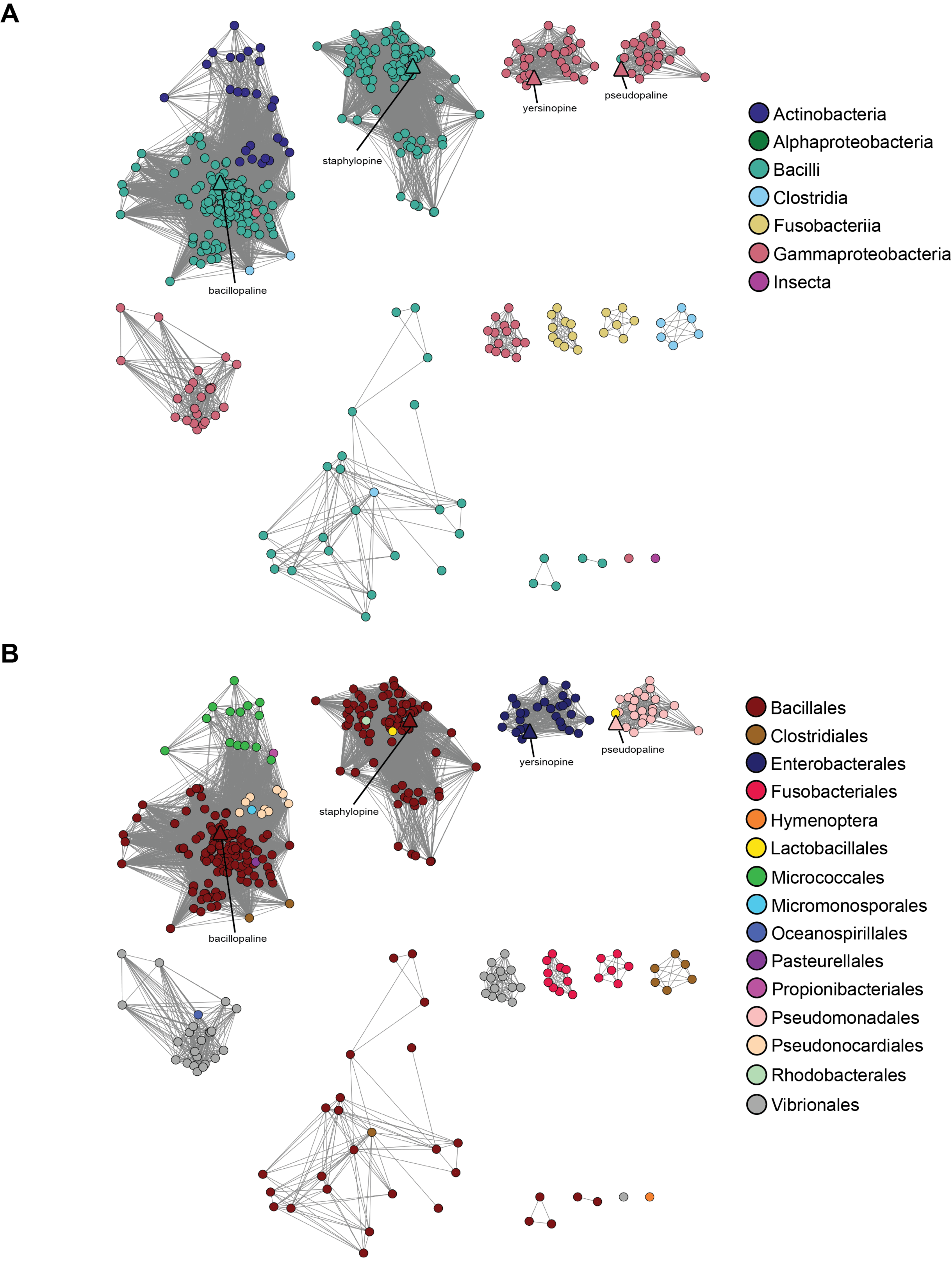

Supplement: FIG S2 [file mSystems.00554-20-sf002.tif]

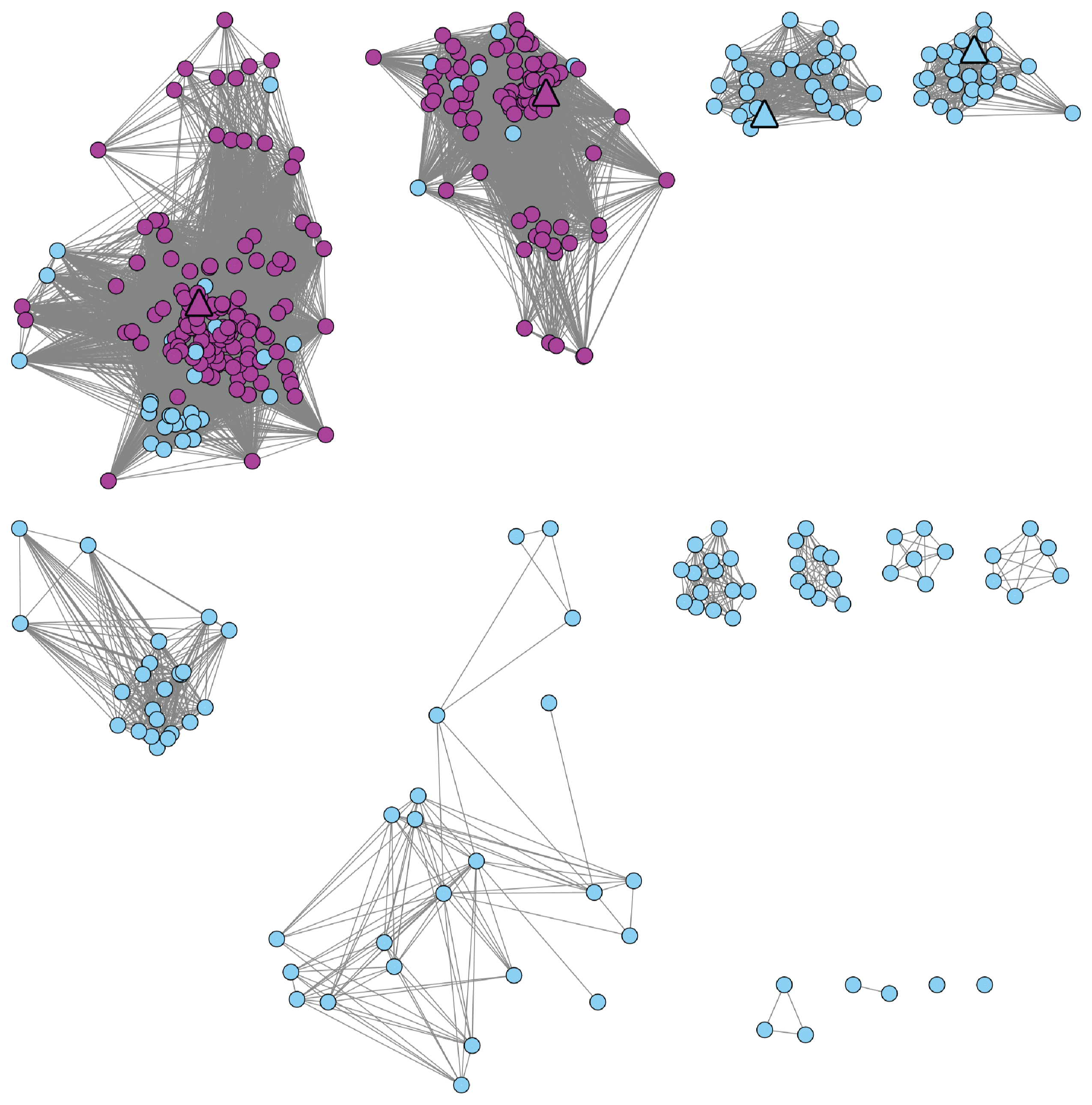

Supplement: FIG S3 [file mSystems.00554-20-sf003.tif]
